# Spontaneously Extruded Osteoma of the External Auditory Canal

**DOI:** 10.1155/crot/9984450

**Published:** 2026-01-28

**Authors:** Kristen L. Zayan, Tracy Cheng, Andrew A. McCall

**Affiliations:** ^1^ Department of Otolaryngology Head and Neck Surgery, University of Pittsburgh Medical Center, Pittsburgh, Pennsylvania, USA, upmc.com; ^2^ Department of Otolaryngology Head and Neck Surgery, Stanford University, Stanford, California, USA, stanford.edu; ^3^ Department of Otolaryngology Head and Neck Surgery, University of California Los Angeles, Los Angeles, California, USA, ucla.edu

**Keywords:** case report, exostosis, external ear canal mass, osteoma

## Abstract

Osteomas of the external ear canal are rare but benign bony growths. We present a case report of an external auditory canal osteoma in a 74‐year‐old female that spontaneously extruded from the ear. The patient was evaluated after the lesion dislodged from her ear canal without physical manipulation or operative intervention, and she presented the specimen intact. The diagnosis of osteoma was made clinically, radiographically, and pathologically. We propose that certain external ear canal osteomas may resolve spontaneously or be amenable to in‐office removal.

## 1. Introduction

Lesions of the external ear canal (EAC) are uncommon, though the diagnosis involves a wide differential including inflammatory masses, congenital abnormalities, benign or malignant tumors, trauma, cholesteatoma, or cerumen impaction [1]. Of the bony EAC lesions, osteomas and exostoses are most common [1]. Osteomas are a rare but benign growth without a clear cause and are characteristically identified via computed tomography (CT) imaging as unilateral, pedunculated masses originating from the tympanosquamous or tympanomastoid suture line along the bony–cartilaginous junction. Patients often present asymptomatically with EAC lesions. Sometimes, patients may present with occlusive symptoms such as otitis externa, cerumen impactions, or hearing loss [2, 3]. Notably, patients with multiple osteomas should be evaluated for Gardner’s syndrome, which includes the constellation of polyps of the colon and impacted or unerupted teeth, with possible skin and soft tissue tumors [4]. In contrast, exostoses have a broad base on CT, are often multiple, and are more frequently seen bilaterally. Repetitive cold‐water exposure or recurrent episodes of acute otitis externa (AOE) can predispose patients to exostosis formation [5].

Patients with concern for an ear canal lesion often initially seek evaluation by an otolaryngologist for recurrent AOE, otalgia, hearing loss, or tinnitus [6, 7]. Concern for a bony lesion of the EAC may be identified via history and physical examination as well as radiographically via CT. Osseous EAC lesions may be found incidentally, though overall there are limited epidemiological data of such occurrences [8]. It is recommended that patients are evaluated by a subspecialist for findings suggestive of an EAC lesion [9]. Treatment for benign, bony EAC lesions depends on patient presentation: These lesions can be observed if patients have no presenting symptoms. However, EAC osteomas may require surgical excision if symptomatic, or if there is a threat of secondary issues such as the development of recurrent infection or cholesteatoma [2, 10–12].

## 2. Case Presentation

A 74‐year‐old female with a history of asthma, hyperlipidemia, non–insulin‐dependent Type 2 diabetes mellitus (DM), and polymyalgia rheumatica on low dose daily steroids was referred to the otolaryngology–neurotology clinic for the evaluation of otalgia with concern for a left‐sided ear mass. The patient had a known left ear lesion for multiple years prior to evaluation. The lesion was first identified incidentally on a noncontrast CT of the head 11 years earlier, which was performed for concern about headaches. Six years later, a dedicated noncontrast CT image of the temporal bones was obtained for further evaluation of the previously identified EAC lesion, which reported a 1.0 × 0.4 × 0.7‐cm left bony canal lesion, “concern for exostosis,” per the radiology report (Figure 1). Two months prior to evaluation at our tertiary clinic, she had another head CT (however, not a dedicated CT of the temporal bones), which showed similar dimensions of the osteoma. Her prior workup included an audiology evaluation 8 months prior, which showed bilateral mild‐to‐moderate sensorineural hearing loss (Figure 2). She ultimately sought neurotology evaluation for further evaluation of intermittent otalgia and a known history of an ear mass. One week prior to her appointment, the mass spontaneously extruded from her left EAC. She denied any trauma or digital manipulation of the auricle or ear canal. The patient overall experienced resolution of her symptoms, including subjective hearing improvement and relief of otalgia.

**Figure FIGURE 1:**
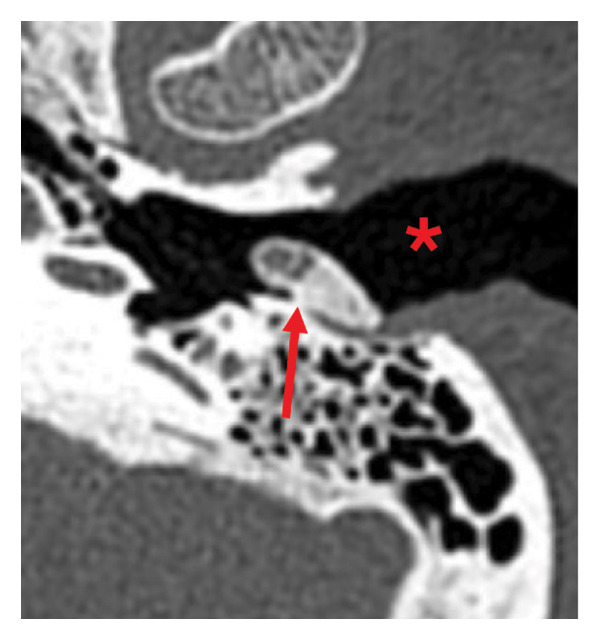
Axial CT of the left temporal bone from 6 years prior to patient presentation, depicting a pedunculated bony lesion within left EAC consistent with EAC osteoma. The red arrow indicates the stalk of the osteoma. The asterisk (∗) denotes the lateral and cartilaginous portion of the EAC.

**Figure FIGURE 2:**
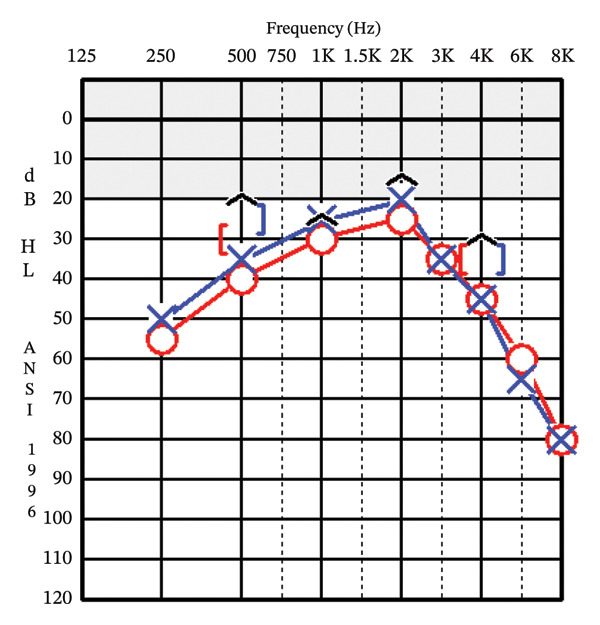
Patient audiogram prior to spontaneous extrusion of the left EAC osteoma showed bilateral symmetric sensorineural hearing loss with word recognition scores of 92% bilaterally. No air bone gap was noted on the left (blue line) despite the patient’s report of subjective hearing loss on the left.

Upon clinical examination, the patient’s left ear revealed intact canal wall skin with no evidence of ulceration or bony canal abnormality. There was a mild amount of blood crusting along the anterior medial canal wall. The tympanic membrane (TM) appeared intact on the left side, with an aerated middle ear (Figure 3). No pathologic changes were noted on the contralateral side. The patient brought the mass with her, and it was noted to be a solid, black‐colored lesion, measuring 1.2 × 0.9 × 0.5 cm (Figure 4). Repeat audiology evaluation was deferred at the patient’s request. Pathology determined the left ear mass to be an osteoma (Figure 5).

**Figure FIGURE 3:**
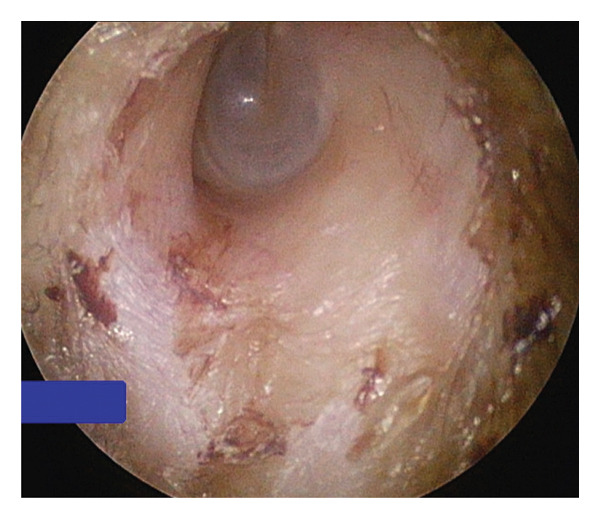
Endoscopic view of the left EAC after spontaneous extrusion of osteoma reveals minimal cerumen and anterior–superior crusting without evidence of significant canal trauma. The tympanic membrane was intact, and there was no middle ear effusion.

**Figure FIGURE 4:**
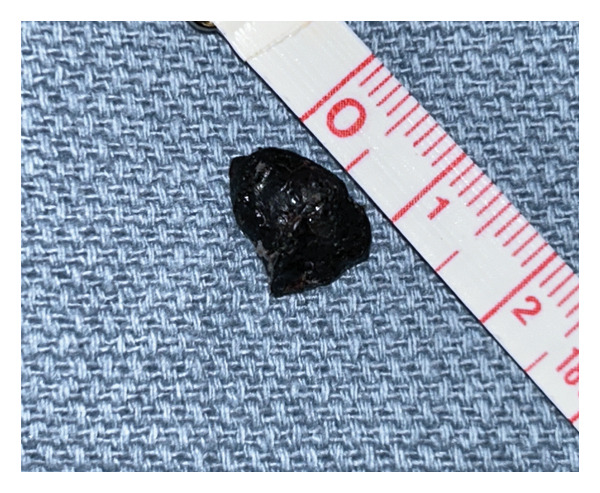
Gross patient specimen of 1‐cm left EAC lesion which spontaneously extruded. The shown measuring tape depicts centimeters (cm).

**Figure FIGURE 5:**
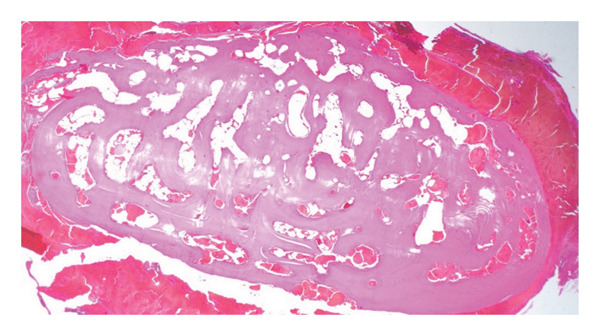
Histopathology with hematoxylin and eosin staining (magnification 20×). Specimen is composed of predominantly sclerotic lamellar bone with surrounding organized hemorrhage, favoring a diagnosis of osteoma. Image courtesy of Dr. Raja R. Seethala, MD, UPMC Department of Pathology and Laboratory Medicine.

## 3. Discussion

This case identifies a unique presentation of an EAC osteoma that had been present for at least 11 years, which spontaneously extruded without manipulation or surgical intervention. Osteomas are slow‐growing and frequently asymptomatic benign neoplasms. EAC osteomas arise from the tympanomastoid or tympanosquamous suture lines of the bony canal. Histologically, osteomas can be compact, spongy, or mixed with lamellated bone with minimal osteocytes, appearing as mature bone. They are covered by periosteum and squamous epithelium, which may appear as healthy EAC skin on binocular microscopic or otoscopic evaluation [8]. Osteomas of the temporal bone are overall rare, though they are most commonly found within the EAC portion [13].

Treatment of osteomas within the EAC varies with each patient, though definite treatment has historically been surgical excision [9]. Observation with conservative measures may be warranted for those with limited symptoms: aural toileting to limit possible sequelae such as cerumen impaction, recurrent infection, conductive hearing loss, or cholesteatoma [9, 12, 14]. As EAC osteomas grow, they can entrap cerumen medial to the lesion, limit drainage of the canal or the ability of otologic drops to reach the TM, and disrupt sound transmission to the TM [3]. Consequently, though histologically benign, osteomas can be locally destructive, particularly when cholesteatomas form and erode surrounding structures [14, 15]. Surgical excision of an EAC osteoma can be performed via transcanal, postauricular, and endaural approaches microscopically or endoscopically, depending on the size and location of the base of the lesion [6, 16, 17].

Interestingly, the patient in our case presentation had this known EAC lesion for at least 11 years, as evidenced by a historical CT of the head exam. She reported gradual progression of otalgia and hearing loss, which ultimately led to her evaluation at a tertiary level neurotology clinic. Mere days before presenting to our clinic, the lesion spontaneously extruded from the patient’s EAC, with complete resolution of her symptoms. Examination revealed a normal appearing ear canal without residual stalk or evidence of trauma. There is no clear etiology of how this lesion spontaneously extruded from her ear. While the bony stalks of osteomas can be thin, removal classically involves an operative procedure, often with extensive chisel or drilling of the bone [18–20]. Surgical removal of EAC osteomas is well tolerated by patients, and they can be performed microscopically or endoscopically, transcanal or retroauricular [18]. Removal of EAC lesions involves careful elevation and preservation of the canal skin to perform canalplasty and fully remove the stalk of the osteoma [20]. Interestingly, in this case, the skin was intact and there was no evidence of residual stalk or bony abnormality. It is possible that the osteoma fully detached well before it extruded from her ear; however, we cannot determine how long prior to extrusion this may have occurred.

Investigation of the patient’s medical history identified multiple comorbidities, which we hypothesize could have predisposed her to poor bone health and put her at risk for extrusion of the osteoma. While insulin‐dependent, Type 1 DM is more classically associated with risk for fractures, there are studies identifying Type 2 DM with a risk ratio (RR) as high as 1.87 for fractures [21, 22]. Prior to evaluation in our clinic, her hemoglobin A1c was 8.1, from May 2023. Additionally, she had been on low‐dose corticosteroids, prednisone 2.5 mg daily for her polymyalgia rheumatica, and it is well supported that chronic steroids may lead to osteoporosis [23]. To our knowledge and from chart review, our patient had no known osteoporosis or bone‐related metabolic disorders, though medical history should be considered in this situation: We propose that this may be partially responsible for spontaneous extrusion of this patient’s EAC osteoma.

Her improvement in symptoms after extrusion of the lesion supports the concept of EAC osteoma removal in symptomatic patients. There are no other reported cases of spontaneously resolving ear canal osteomas in the literature. To our knowledge, there are also no reports in the literature of in‐office transcanal osteoma removal. The outcomes of this case suggest that this can be attempted for lateral lesions with a very narrow bony stalk, in patients who are agreeable and can tolerate an attempt at removal. In summary, surgery may not be required for all patients with EAC osteomas. Alternatives to treatment include conservative management with cerumenectomy as needed, use of hearing amplification devices for associated hearing loss, and close observation for the development of recurrent infections or cholesteatoma. Furthermore, osteomas may spontaneously resolve over time as demonstrated in this case report.

## Funding

This research did not receive any specific grant from funding agencies in the public, commercial, or not‐for‐profit sectors.

## Consent

Informed consent was obtained. Privacy rights of the patient have been observed.

## Conflicts of Interest

The authors declare no conflicts of interest.

## Data Availability

The data that support the findings of this study are available upon request from the corresponding author. The data are not publicly available due to privacy or ethical restrictions.
